# Association between obstructive sleep apnoea and breast cancer: The Korean National Health Insurance Service Data 2007–2014

**DOI:** 10.1038/s41598-019-55551-7

**Published:** 2019-12-13

**Authors:** Ji Ho Choi, Jae Yong Lee, Kyung Do Han, Young Chang Lim, Jae Hoon Cho

**Affiliations:** 1 Department of Otorhinolaryngology-Head and Neck Surgery, Soonchunhyang University College of Medicine, Bucheon Hospital, Bucheon, Korea; 2 Department of Biostatistics, Catholic University College of Medicine, Seoul, Korea; 3 Department of Otorhinolaryngology-Head and Neck Surgery, College of Medicine, Konkuk University, Seoul, Korea

**Keywords:** Neurology, Risk factors

## Abstract

Some studies have argued that obstructive sleep apnoea (OSA) increases the risk of breast cancer. However, the results are often conflicting. This study aimed to investigate associations between OSA and breast cancer incidence using the Korea National Health Insurance Service (KNHIS) database. This retrospective cohort study analyzed data from the KNHIS database. A total of 45,699 women (≥20 years of age) newly diagnosed with OSA between 2007 and 2014 were included. The control groups were a 5-fold, age-matched women who had not been diagnosed with OSA. Mean follow-up duration was 3.7 ± 2.3 years. The primary endpoint was newly diagnosed breast cancer. The breast cancer hazard ratio (95% confidence interval) was calculated for patients with OSA and compared with that of the control group. The incidence of breast cancer among patients with OSA was significantly higher than that among the controls (1.20 [1.04–1.39]). In particular, the incidence of breast cancer was higher among patients aged ≥65 years (1.72 [1.10–2.71]). The result suggests that OSA may be a risk factor for breast cancer in women.

## Introduction

According to the American Academy of Sleep Medicine (AASM) guidelines, obstructive sleep apnoea (OSA) is considered a chronic disease, and long-term multidisciplinary management is recommended^1^. OSA is a common disorder characterised by repeated respiratory disturbances due to total or partial obstruction of the upper airway during sleep^2^. The prevalence of OSA in Korea is 4.5% in men and 3.2% in women, similar to that in western countries^3,4^. OSA is associated with various harmful mechanisms (e.g., intermittent hypoxia, hypercapnia, increased sympathetic activity, sleep fragmentation, and variation of intrathoracic pressure)^5^. Eventually, OSA causes many symptoms (e.g., excessive daytime sleepiness, reduced concentration, memory loss, decreased libido, non-refreshing sleep, insomnia, and morning headache) and serious consequences (e.g. hypertension, coronary artery disease, arrhythmia, heart failure, insulin resistance, and cerebrovascular disease)^6^. Furthermore, recent clinical investigations propose that OSA is associated with diverse cancers^7,8^. According to a study by Nieto *et al*., OSA was associated with total and cancer mortality even after adjusting for age, sex, body mass index, and smoking. Moreover, as the apnea-hypopnea index increased, the association increased. In severe cases, cancer mortality increased 4.8 times compared to the normal group^7^.

Breast cancer is the most common cancer in women and the second most commonly diagnosed malignancy worldwide^9^. Further, breast cancer is internationally accepted as the fifth most common cause among the total cancer deaths^10^. Similarly, breast cancer is the second most common cancer diagnosed in women, and the incidence of breast cancer has elevated steadily for decades in Korea^11^. It is known that hypoxia is strongly involved in oncogenesis, tumour angiogenesis, and metastasis^12^. Recent basic researches suggest that intermittent hypoxia, but not chronic hypoxia, may be related to the promotion of breast cancer survival and metastatic growth^13,14^.

In addition, clinical researches have reported that OSA may be a risk factor for the occurrence of breast cancer in women^15–18^. However, other studies suggest that incidence of breast malignancy in OSA patients is not significantly higher than that in control individuals^19,20^. Therefore, the purpose of this clinical investigation is to evaluate associations between OSA and the incidence of breast cancer based on the Korea National Health Insurance Service (KNHIS) database.

## Materials and Methods

### Data source

The KNHIS covers the entire Korean population^21^. Both outpatient and inpatient claims are reviewed by the KNHIS and include data on diagnoses, procedures, prescription records, demographic information, and direct medical costs. The KNHIS identifies its members by their Korean Resident Registration Number, which removes the potential risk of duplication or omission when accessing the data. The KNHIS database manages claims using the Korean Classification of Disease, sixth edition, a modified version of the International Classification of Diseases, 10^th^ edition (ICD-10), adapted for the Korean healthcare system. Any researcher can use the KNHIS data if the study protocols are approved by the official review committee. The KNHIS data from 2002 to 2017 is available now.

### Study population and design

**Figure 1:**
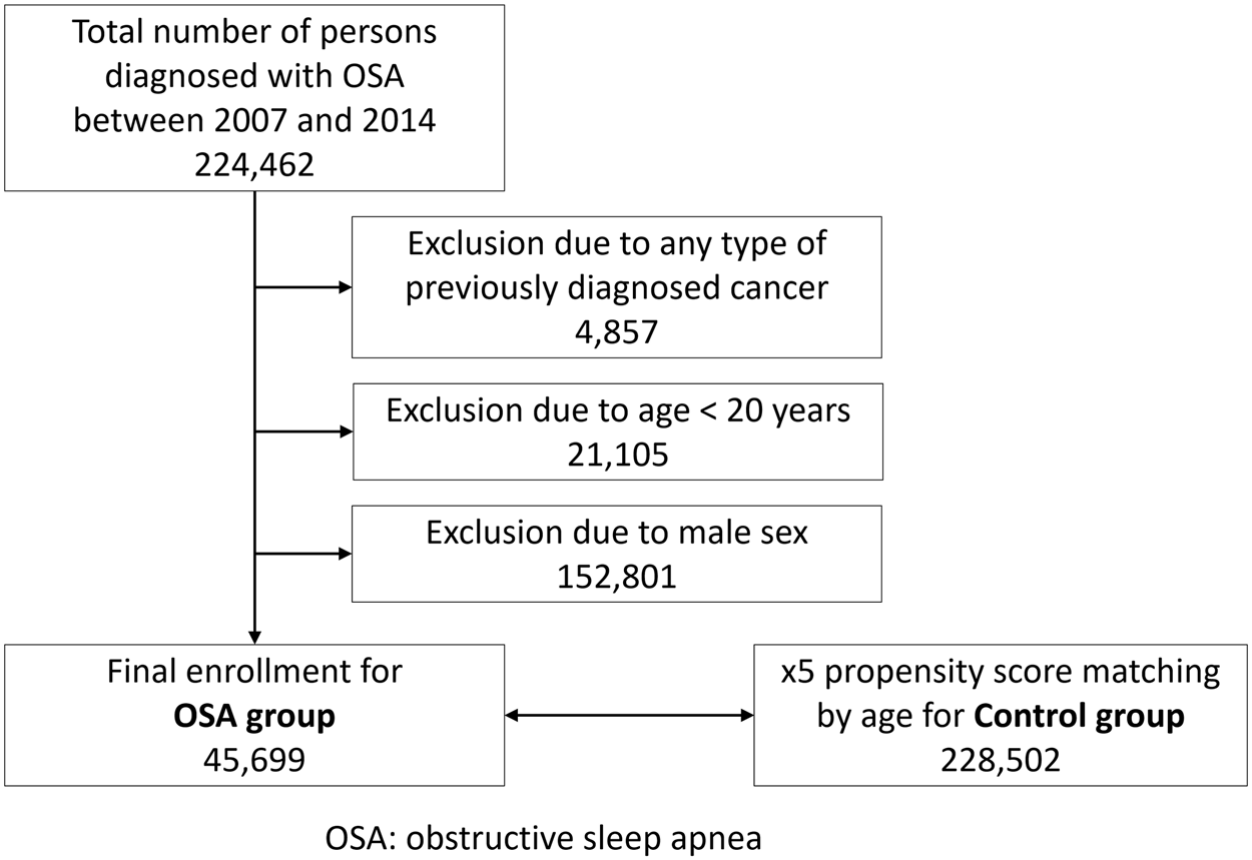
Enrollment flowchart.

We defined the OSA group as that including women aged ≥20 years with newly diagnosed OSA (G47.30) between 2007 and 2014. We selected a 5-fold, age-matched control group that had not been diagnosed with OSA. People diagnosed with any types of cancer within five years prior to enrollment were excluded. Therefore, data were available since 2007. In addition, to ensure follow-up of at least 3 years from study participation, only data before 2014 were used. Breast cancer that occurred within one year of enrollment was excluded from the analysis because breast cancer that occurred before or immediately after OSA diagnosis cannot be related to OSA. The primary endpoint of this study was the incidence of newly diagnosed breast cancer. A flowchart showing the enrolment process for this study has been presented. (Fig. 1).

### Data collection

We collected the following baseline data from the KNHIS database: age (years) and income level (four quintiles). Data on comorbidities including diabetes, hypertension, dyslipidaemia, stroke, chronic obstructive pulmonary disease, and ischemic heart disease were also collected. These comorbidities are defined in the Table 1.

**Table 1:** Working definitions derived from insurance claims data.

Disease	Working definition
Obstructive sleep apnea	at least one claim under ICD-10 code G47.3
Breast cancer	at least one claim under ICD-10 code C50 and registered as a cancer patient in the National Medical Expenses Support Program
Diabetes	at least one claim per year for the prescription of anti-diabetic medication under ICD-10 code E11-14
Hypertension	at least one claim per year for the prescription of anti-hypertensive medication under ICD-10 code I10-13 or I15
Dyslipidemia	at least one claim per year for the prescription of anti-dyslipidemic medication under ICD-10 code E78
Stroke	at least one claim under ICD-10 code I63 or I64
COPD	at least one claim under ICD-10 code J41, J42, J43, or J44
IHD	at least one claim under ICD-10 code I20, I21, I22, I23, I24, or I25

ICD: international classification of diseases, COPD: chronic obstructive pulmonary disease, IHD: ischemic heart disease.

### Statistical analysis

Data are presented as the mean ± standard deviation (SD) for age and as proportions for the remaining categorical variables. Comparisons between two groups were made using the Student’s t test for continuous variables or Chi-squared test for categorical variables. A Kaplan-Meier plot without covariance correction is presented to analyse the risk of breast cancer according to the presence or absence of OSA. The incidence of breast cancer was calculated by dividing the number of events by the product of number of persons at risk and time. To determine the hazard ratio of OSA on the relative incidence of breast cancer, the Cox proportional hazards model was used after stratifying for covariates including income level and diabetes, hypertension, and dyslipidaemia statuses. Two different models were applied. Model 1 was not adjusted by any covariate. Model 2 was adjusted for income level and diabetes, hypertension, and dyslipidaemia statuses. In addition, differences in the hazard ratio were also analysed according to age groups. The results are presented as the mean, and 95% confidence interval (95% CI). All statistical analyses were performed using SAS version 9.4 (SAS Institute, Cary, NC, USA) and R version 3.2.3 (The R Foundation for Statistical Computing, Vienna, Austria).

### Ethical approval

The study was exempted from the requirement for informed consent by the Institutional Review Board of Soonchunhyang University Hospital because of the use of publicly available data (SCHBC 2019-08-018). All experimental protocols were approved by the same Institutional Review Board. And all methods were carried out in accordance with relevant guidelines and regulations.

## Results

A total of 49,570,064 subjects enrolled in the KNHIS in 2007. The research data for the first year are available, and the numbers are comparable with that of each subsequent year up to 2014. Between 2007 and 2014, there were 45,699 female patients who were newly diagnosed with OSA. A total of 228,502 subjects were selected as controls (Fig. 1). An enrolment flowchart is summarised in Fig. 1.

### Comparison between the OSA and control groups

The ages of the two groups were exactly matched, but the other characteristics were somewhat different. The income level of the control group was slightly lower, and all other comorbidities were common in the OSA group. The results are summarized in Table 2.

**Table 2:** Demographics of patients with OSA and controls.

	OSA		Controls		*p* value
	N	%	N	%	
Total number	45,699	100.0	228,502	100.0	
Follow-up duration (years)	3.7 ± 2.3		3.7 ± 2.3		1.000
Total person-year	170,457		852,312		
Mean age (years)	48.7 ± 14.2		48.7 ± 14.2		1.000
Age ≥65 years	5,835	12.8	29,129	12.8	1.000
Income in the lowest quintile	10,348	22.6	57,498	25.2	<0.001
Diabetes	3.545	7.8	13,826	6.1	<0.001
Hypertension	11,577	25.3	40,393	17.7	<0.001
Dyslipidemia	9,029	19.8	27,351	12.0	<0.001
Stroke	2,750	6.0	6,662	2.9	<0.001
COPD	8,836	19.3	28,267	12.4	<0.001
IHD	817	1.8	1,798	0.8	<0.001

OSA: obstructive sleep apnoea, COPD: chronic obstructive pulmonary disease, IHD: ischemic heart disease.

### Hazard ratio of the OSA group compared with that of the control group with regard to breast cancer incidence

The Cox proportional hazards model showed that the hazard ratio for breast cancer incidence in the OSA group was significant for both models. The hazard ratio based on Model 1, which was not adjusted, was 1.19 (95% CI 1.03–1.37), and it was nearly similar after adjusting for various comorbidities in Model 2. The age-related risk was highest at 1.72 (95% CI 1.10–2.71) in the age group of ≥65 years, and lowest at 1.05 (95% CI 0.69–1.59 for those aged 20 ≤ and <40 years. These results are summarized in Tables 3 and 4.

**Table 3:** OSA hazard ratio for the incidence of breast cancer.

	N	Event	Rate	Model 1	Model 2
Controls	228,502	955	1.12	1	1
OSA	45,699	227	1.33	1.19 (1.03–1.37)	1.20 (1.04–1.39)

Model 1: not adjusted; Model 2: adjusted by income level and diabetes, hypertension, and dyslipidaemia statuses.

OSA: obstructive sleep apnoea.

Hazard ratio was represented with 95% confidence interval (95% CI).

**Table 4:** OSA hazard ratio for the incidence of breast cancer by age group.

Age (years)	20 ≤ age ≤ 40	40 ≤ age ≤ 65	65 ≤ age
Controls	1	1	1
OSA	1.05 (0.69–1.59)	1.15 (0.98–1.36)	1.72 (1.10–2.71)

OSA: obstructive sleep apnoea.

Hazard ratio was represented with 95% confidence interval (95% CI).

### The Kaplan-meier plot between the OSA group and controls

**Figure 2:**
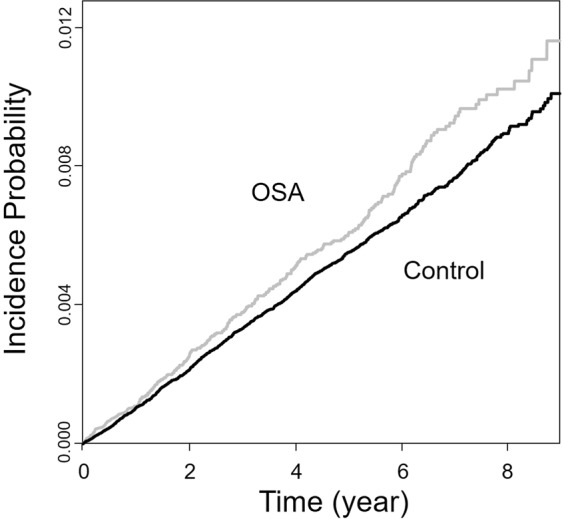
Kaplan-Meier plot of the incidence of breast cancer among patients with OSA. Breast cancer occurs more frequently in the OSA group than in the control group. OSA: obstructive sleep apnoea.

The Kaplan-Meier plot shows the incidence of breast cancer without adjustment. Breast cancer occurred more frequently in the OSA group than in the control group (Fig. 2).

## Discussion

The results of the current study suggest that breast cancer may be associated with OSA, based on the cohort data analysis of a nationwide population. In this study, breast malignancy incidence in OSA patients was significantly higher than that in control individuals. Moreover, there was a significant increase in breast cancer incidence in OSA patients aged ≥65 years.

Some clinical researches have reported that OSA elevates the risk of cancer, including that of the breast^15–18^. The Spanish sleep network group evaluated the association between OSA and increased incidence of malignancy in a large multi-institutional cohort and found that elevated nocturnal respiratory disturbance or hypoxia were associated with an elevated incidence of malignancy, including that of the breast^15^. Chang *et al*. demonstrated that the OSA patient group had a twofold elevated risk of breast malignancy (95% CI 1.06–4.12) compared with the control group after adjusting for potential confounders^16^. Sillah *et al*. argued that the overall malignancy incidence of patients with OSA was approximately 26% higher than that of the general population^17^. More specifically, a significantly increased incidence of breast cancer was shown among patients with OSA, especially the older population^17^. Brenner *et al*. confirmed that severe OSA patients aged <45 years have a significantly higher overall cancer incidence compared with the general population (hazard ratio, 95% CI 1.12–12.45)^18^. All these results of earlier investigations are in close agreement with those of the present study that revealed an association between OSA and breast cancer.

A few studies, however, showed that OSA is not associated with an increase of breast cancer incidence^19,20^. Gozal *et al*. reported that the incidence of breast malignancy (hazard ratio 0.96, 95% CI 0.93–0.99) was lower in patients with OSA when compared with control group^19^. Campos-Rodriguez *et al*. concluded that there are no associations between OSA and the aggressiveness of breast cancer^20^.

It is still unclear how OSA affects the incidence of breast cancer. However, there is a hypothesis that explains the link between OSA and cancer development. As already known, intermittent hypoxia is a very critical mechanism related with the OSA symptoms and complications. Liu *et al*. evaluated the effects of intermittent hypoxia on the proliferation and migration of human breast cancer cells^13^. Their study suggests that a significant increase in the migration of cancer cells was caused by intermittent hypoxia. Chen *et al*. documented that intermittent hypoxia leads to some crucial changes (e.g., gene expression, cellular heterogeneity, and paracrine signalling) that promote cancer cell survival, colonisation, and metastatic growth^14^. Sleep fragmentation also plays an important role in the pathogenesis of OSA. Hakim *et al*. verified the hypothesis that sleep fragmentation facilitates tumour development and invasiveness using tumour cells and model^22^. Their findings suggested that sleep fragmentation can promote tumour growth and advancement via the recruitment of tumour-associated macrophages and pro-inflammatory TLR4 signalling pathway.

In this study, we observed a tendency to increase the risk of breast cancer as the age of OSA patients increased. The exact cause is unknown, but it is presumed that older groups are affected by intermittent hypoxia and sleep disruption for longer periods of time. Additionally, the prevalence and severity of OSA in postmenopausal women were significantly higher than those in premenopausal women, which may also be the reason^23,24^.

The goal of this study was to appraise the association between OSA and the incidence of breast cancer using a large nationwide (KNHIS) database. However, there are several limitations to this study. (1) The KNHIS data do not include any information about the validity of the diagnosis and OSA severity. The OSA group was defined based only on the diagnostic code of the claim data. Given that the prevalence of OSA is quite high, the control group will also contain a large number of patients with OSA. (2) There were not enough data available on confounders involved in both OSA and breast cancer, including body mass index, lifestyle, family history, exercise, cigarette smoking, alcohol drinking. Without data on these confounders, it is impossible to clearly establish a causal relationship. (3) The results of this study may not be representative of all races because only Korean data were used. (4) The calculation of person-years at risk did not consider death. OSA increases mortality substantially. Therefore, the person-years may have been over-estimated for participants who did not develop breast cancer but died prior to end of follow up.

## Conclusion

OSA may be a risk factor for breast cancer in women.
